# Fatty Acid Composition and Levels of Selected Polyunsaturated Fatty Acids in Four Commercial Important Freshwater Fish Species from Lake Victoria, Tanzania

**DOI:** 10.1155/2014/712134

**Published:** 2014-12-25

**Authors:** Agnes Robert, Prosper Mfilinge, Samwel M. Limbu, Chacha J. Mwita

**Affiliations:** ^1^National Museum of Tanzania, P.O. Box 511, Dar es Salaam, Tanzania; ^2^Department of Aquatic Sciences and Fisheries, University of Dar es Salaam, P.O. Box 35064, Dar es Salaam, Tanzania

## Abstract

Fatty acids (FAs) particularly *ω*3 and *ω*6 polyunsaturated fatty acids (PUFAs) play important role in human health. This study aimed to investigate the composition and levels of selected *ω*3 PUFAs in four commercial fish species, Nile perch (*Lates niloticus*), Nile tilapia (*Oreochromis niloticus*), *Tilapia zillii*, and dagaa (*Rastrineobola argentea*) from Mwanza Gulf in Lake Victoria. The results indicated that 36 types of FAs with different saturation levels were detected. These FAs were dominated by docosahexaenoic (DHA), eicosapentaenoic (EPA), docosapentaenoic (DPA), and eicosatetraenoic acids. *O. niloticus* had the highest composition of FAs (34) compared to *L. niloticus* (27), *T. zillii* (26), and *R. argentea* (21). The levels of EPA differed significantly among the four commercial fish species (*F* = 6.19,  *P* = 0.001). The highest EPA levels were found in *R. argentea* followed by *L. niloticus* and *O. niloticus* and the lowest in *T. zillii*. The DPA levels showed no significant difference among the four fish species studied (*F* = 0.652,  *P* = 0.583). The study concluded that all four commercial species collected from Mwanza Gulf are good for human health, but *R. argentea* is the best for consumption because it contains higher levels of *ω*3 FAs, mainly EPA.

## 1. Introduction

Freshwater fishes contain saturated fatty acids (SAFAs), monounsaturated fatty acids (MUFAs), and long-chain polyunsaturated fatty acids (PUFAs) that have significant role in human health. Polyunsaturated fatty acids (PUFAs) are particularly important due to their ability to prevent cardiovascular disease, psychiatric disorders, and some other illnesses such as atherosclerosis, thrombogenesis, high blood pressure, cancer, and skin diseases [1]. PUFAs are commonly categorized into two main groups omega 3 (*ω*3) and omega 6 (*ω*6) depending on the position of the first double bond from the methyl end group of the fatty acid [2]. The main *ω*3 PUFAs playing important role in human health include *α*-linolenic acid (ALA), docosahexaenoic acid (DHA), eicosapentaenoic acid (EPA), and docosapentaenoic acid (DPA) and *ω*6 PUFAs include linoleic acid (LA) and arachidonic acid (ARA) [2]. These PUFAs are not synthesized in the human body and therefore inclusion in human diets is a necessity [3]. Ultimately, it is important to take a proactive approach to ensure sustained access and uptake of PUFAs for proper maintenance of our health [4].

The chief sources of PUFAs in human diet include fish [1]. However, different species have variation in their FA composition and levels [4]. The variation in FA of fish is due to diet consumed, reproductive cycle, temperature, season, and geographical location [4–7]. Nile perch* Lates niloticus*, Nile tilapia* Oreochromis niloticus*,* Tilapia zillii*, and* Rastrineobola argentea* fishes constitute great resources for communities living around Lake Victoria [8]. The fishes are widely distributed throughout the lake representing the most fished species for human consumption. They have significant commercial and ecological roles in the lake ecosystem [9] and aquaculture potential particularly for* Oreochromis niloticus* and* Tilapia zillii* [10, 11]. The nutritional quality of these species in the area is important for rural communities since they are crucial diets and provide livelihoods subsequently influencing community health.

Previous studies on FAs of fish species in Lake Victoria have concentrated on growth stages and general proximate analysis particularly in Kenyan and Ugandan waters of Lake Victoria. Kizza et al. [12] analyzed the lipid content and FA profiles of* Lates* species whereas Turon et al. [13] assessed the FAs composition of oil extracted from the head of Nile perch. The FA compositions of muscle and heart tissue of Nile perch,* Lates niloticus*, and Nile tilapia,* Oreochromis niloticus*, in Lakes Victoria and Kioga have been investigated by Kwetegyeka et al. [14]. Mwanja et al. [15] characterized the fish oils of mukene (*Rastrineobola argentea*) of Nile basin waters, Lake Victoria, Lake Kyoga, and the Nile River. Namulawa et al. [16] established the FA profiles of the eggs and juvenile muscle of Nile perch (*Lates niloticus*). The use of FA profile of the polar fraction as a taxonomic marker for Nile perch* Lates niloticus*, Nile tilapia* Oreochromis niloticus*, marbled lungfish* Protopterus aethiopicus*,* Bagrus docmak*, and African catfish* Clarias gariepinus* was compared by Kwetegyeka et al. [17]. The effects of heavy metal pollution on *ω*3 PUFAs levels in tilapia fish from Winam Gulf of Lake Victoria were explored by Muinde et al. [18]. The proximate composition of* Rastrineobola argentea* (Dagaa) of Lake Victoria-Kenya has been analyzed by Ogonda et al. [19].

Although these studies have been conducted in Lake Victoria, none was carried out in the Tanzanian waters. Moreover, there is lack of information on the composition and levels of PUFAs of freshwater fish species at different trophic levels in Lake Victoria. Limited literature is available on the FA profile of* Tilapia zillii* although it is among the most consumed fish around Lake Victoria. The only data available in this species is that from Olagunju et al. [20] in Nigeria who analyzed the nutrient composition of* Tilapia zillii*. The present study investigated the types and levels of *ω*3 PUFAs in four different species, Nile perch (*Lates niloticus*), Nile tilapia (*Oreochromis niloticus*),* Tilapia zillii*, and dagaa (*Rastrineobola argentea*) in Mwanza Gulf of Lake Victoria.

## 2. Materials and Methods

### 2.1. Collection of Fish Samples

A total of 48 individual species of* Lates niloticus, Oreochromis niloticus, Tilapia zillii*, and* Rastrineobola argentea* were collected from Mwanza Gulf and morphologically identified using keys given by Eccles [21] and Skelton [22]. Ten individuals were collected per species. After collection each sample was stored in plastic bags and preserved by using dry ice while still in the boat and later frozen at −20°C. Frozen samples were shifted by airplane to the Zoology Laboratory at the University of Dar es Salaam for analysis.

### 2.2. Sample Preparation and Lipid Extraction

In the laboratory, the fish samples were thawed to remove the ice for easy cutting of tissues. Each individual fish was cut, starting from the upper part below the dorsal fin down to the abdomen. Sample tissues were washed in water to remove blood and dried with a tissue paper to remove excess water. For each sample a weight between 10 and 20 g was grinded to soften the muscles. Precautions were taken to avoid contamination.

Extraction of lipid was done by using methanol and chloroform at a ratio of 2 : 1 and mixed by a vortex for 2 minutes. The samples were then stored in a refrigerator for two days to speed up extraction of lipid. Filtration was done using filter paper to separate tissues to obtain filtrate solution. Addition of extra 1 : 1 methanol to chloroform was done to extract the remaining lipids from the tissues. The two layers formed by fish tissues (lipids and aqueous solution) were separated by using a separating funnel to obtain lipids followed by addition of sodium sulphate to remove traces of water from the lipids. Evaporation to remove chloroform was done locally in air conditioned room at 16°C for 24 hours.

### 2.3. Preparation of FA Methyl Esters (FAMEs)

Methylation was done by using concentrated sulphuric acid to obtain FAMEs. Five (5) mg of lipid was suspended in 1 mL toluene prior to derivatization. Then, 2 mLs of methanoic sulphuric acids (1% v/v) was added to each sample in vials and sealed. The samples were heated in a stopper tube at a temperature of 50°C overnight for 16 hours to speed up the reaction. This was followed by addition of 2 mLs of water containing sodium bicarbonate (2%: w/v) to each sample to neutralize the acids. Extractions of product were done by addition of hexane/diethyl ester (1 : 1, by Vol; 2 × 5 m). Evaporation to remove acids was done locally in air conditioned room at 16°C for 48 hours.

### 2.4. Analysis of FAs

The determination of types and levels of omega 3 PUFAs was done by using a gas chromatograph mass spectrometer (GC-MS-QP2010 Ultra), which was equipped with flame ionization detector (FID). 1 *μ*L of FAME in hexane was injected into the GC-MS in a split ratio −1.0. Helium was used as the carrier gas at a flow rate of 2 mL/min. The injector temperature was at 250°C. Temperature was programmed as follows: column oven was set at 90°C, held for 2 minutes, and then increased to 260°C and held for 5 minutes and total time was 41 minutes. The *ω*3 PUFAs (ALA, EPA, and DPA) were identified by comparing their retention time with those of commercial standards.

### 2.5. Statistical Analyses

The data is presented as mean ± standard error and was tested for homogeneity of variances using Levene's test. Then the data was analyzed by using one way analysis of variance (ANOVA) to compare the levels of *ω*3 PUFAs. Tukey's HSD multiple comparisons test was done to evaluate specific differences in the levels of the selected *ω*3 PUFAs in the four fish species. Values with *P* ≤ 0.05 were considered significant. All analyses were done using Statistical Package for Social Science (SPSS) version 20 for windows.

## 3. Results

### 3.1. Composition of FAs in the Four Freshwater Fish

A total of 36 FAs were identified in the four commercial species of fish from Mwanza Gulf of Lake Victoria (Table 1). The unsaturated FAs were relatively many (27) compared to SAFAs (9). The most dominant SAFAs were palmitic acid, pentadecanoic acid, stearic (octadecanoic) acid, tetracosanoic acid, and heptadecanoic acid. Twenty (20) of the 27 unsaturated FAs were PUFAs and 7 were MUFAs. Among the 20 types of PUFAs, the *ω*3 PUFAs were relatively more abundant (8), followed by *ω*6 PUFAs (7). Omega 9 PUFA was recorded only once. The dominant *ω*3 PUFAs were docosatrienoic, docosapentaenoic, docosahexaenoic, eicosapentaenoic, and eicosatetraenoic acids. The principal *ω*6 PUFAs were gamma linolenic and arachidonic acids. The *ω*9 PUFAs and MUFAs were dominated by eicosadienoic acid and oleic acid, respectively.

**Figure 1:**
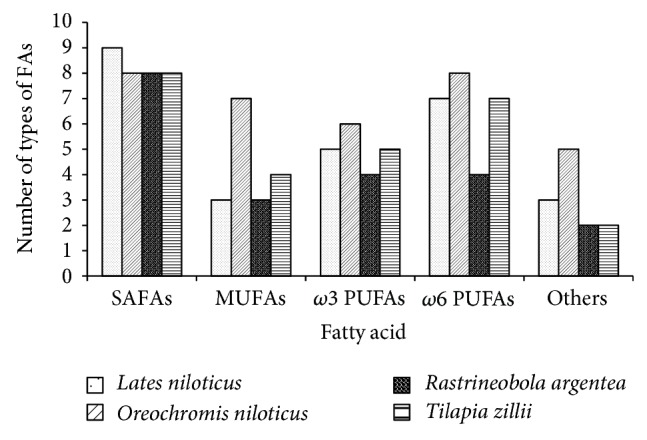
Types of FAs in each of the four commercial fish species studied.

*O*.* niloticus* had comparatively the highest number of FAs particularly in MUFAs, *ω*3 PUFAs, and *ω*6 PUFAs and other unsaturated FAs compared to* L. niloticus*,* T. zillii*, and* R. argentea* (Figure 1). Some of the types of FAs that were found only in* O. niloticus* included hexadecadienoic acid, alpha linolenic acid, 9-tetradecenoic acid, 11-tetradecenoate, and 11, 13-eicosadienoic acid (Table 1).

### 3.2. Types of *ω*3 PUFAs Found in the Four Freshwater Fish Species

The total number of *ω*3 PUFAs detected in the fish samples was eight. The dominant *ω*3 PUFAs in all four sampled commercial fish species were docosatrienoic acid, docosahexaenoic acid, docosapentaenoic acid, eicosapentaenoic acid, and eicosatetraenoic acid (Table 2).

**Figure 2:**
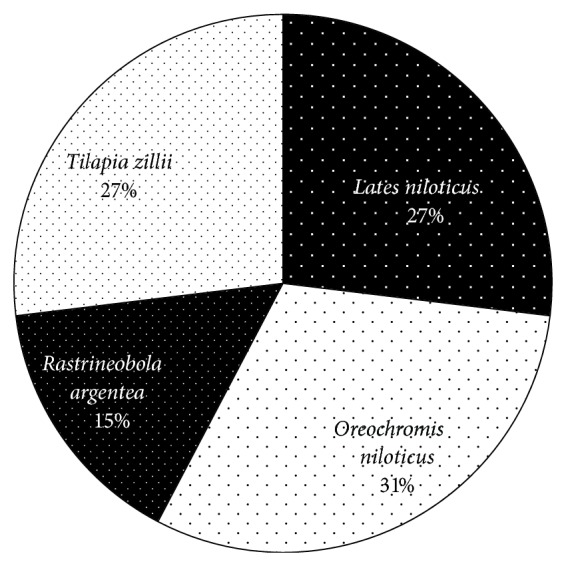
Types of *ω*3 PUFAs in the four commercial fish species.

Similar to types of FAs,* O. niloticus* recorded more types of *ω*3 PUFAs (8 equivalent to 31%) than* L. niloticus* (7),* T. zillii* (7), and* R. argentea* (4) (Figure 2).

### 3.3. Levels of Selected *ω*3 PUFAs Found in the Four Freshwater Fish

**Figure 3:**
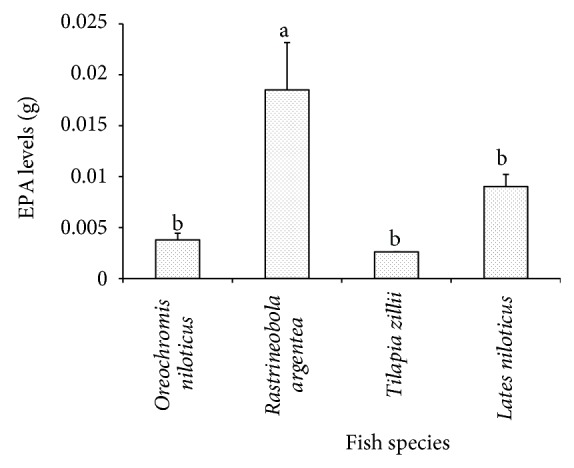
The EPA levels in* R. argentea*,* O. niloticus*,* T. zillii*, and* L. niloticus*.

The levels of EPA for the four freshwater species are shown in Figure 3. The results showed a significant difference in EPA levels among the four commercial fish species (*F* = 6.19, *P* = 0.001). Tukey's HSD multiple comparisons test showed significant higher levels of EPA in* R. argentea* than* O. niloticus* (*P* = 0.001),* T. zillii* (*P* = 0.036), and* L. niloticus* (*P* = 0.009).

**Figure 4:**
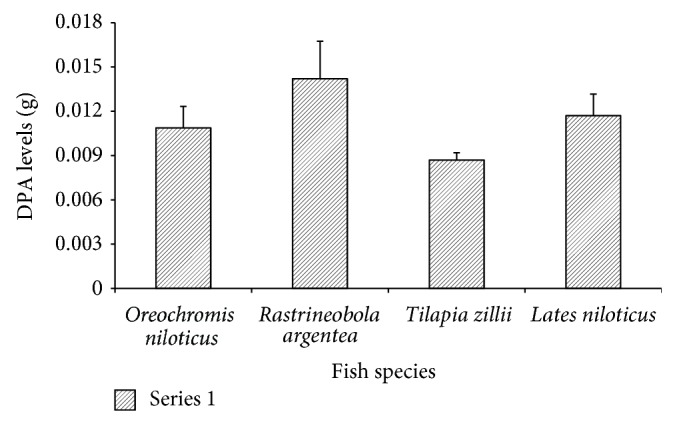
The DPA levels in the four commercial fish species.

The levels of DPA in the four commercial species are shown in Figure 4. The DPA levels were not significantly different among the four commercial species (*F* = 0.652, *P* = 0.583).

## 4. Discussion

This study found 36 types of FAs with different saturation levels in the four commercial fish species. These results are reasonably similar to those obtained by Mohamed and Al-Sabahi [23]. In their study, they obtained 33 FAs of different saturation levels comparable to the present results. Out of the 36 FAs, the saturated SAFAs were 9 (25%) and unsaturated ones were 27 (75%) which included 20 PUFAs and 7 MUFAs. The dominant unsaturated FAs were octadecanoic acid, arachidonic acid, docosapentaenoic acid, eicosapentaenoic and docosahexaenoic acid, and oleic acid. The existence of more unsaturated FAs than saturated FAs in the fish samples is similar to Mwanja et al. [15] who obtained more categories of unsaturated FAs (53.91%) than saturated FAs (46.24%) in* R. argentea*. The dominant unsaturated FAs in this study are similar to those obtained by Zenebe et al. [24], Ugoala et al. [25], Mohamed and Al-Sabahi [23], Osibona [26], Görgün and Akpinar [27], Effiong and Fakunle [28], and Muinde et al. [18]. The more unsaturated FAs than saturated FAs obtained in this study are probably due to their natural ubiquitous occurrence [29]. Freshwater species are known to contain appreciable amount of unsaturated FAs [30] and sometimes more than saturated ones [15, 24].

The dominant *ω*3 PUFAs found in all four commercial fish species were of EPA, DPA, and DHA. This finding is similar to Zenebe et al. [24] and Görgün and Akpinar [27] who described that the most abundant FAs in freshwater species are EPA and DHA. The domination of these *ω*3 PUFAs might be attributed to the feeding habit of the four species. The three species (*R. argentea*,* O. niloticus*, and* Tilapia zillii*) feed lower in the food chain mainly on microalgae (diatoms and dinoflagellates) which are excellent sources of EPA, DPA, and DHA. For example, a study by Mfilinge et al. [31] and Meziane et al. [32] reported that diatoms and dinoflagellates contain higher concentrations of EPA and DHA, respectively, and have been used as markers of diatoms and dinoflagellates in the aquatic food web.* O. niloticus* and* R. argentea* are the major prey of* L. niloticus* in Lake Victoria [33, 34]. Equally,* T. zillii* which has relative similar size and shape is also consumed by* L. niloticus* [35]. Based on this feeding chain, it is more likely that the EPA, DPA, and DHA contained in the herbivorous fish species were transferred to the carnivorous ones via the food chain.

The present results showed that* O. niloticus* had relatively more types of *ω*3 PUFAs than the other species,* T. zillii*,* L. niloticus*, and* R. argentea*. This finding is similar to Ogwok et al. [36] and Muinde et al. [18]. The more types of *ω*3 PUFAs in* O. niloticus* might be attributed to the diverse food items consumed by the fish.* O. niloticus* is an omnivorous fish consuming diverse species of phytoplankton, insects, and juveniles fish. A study by Rumisha and Nehemia [37] reported that* O. niloticus* feeds primarily on Cyanophyta, diatoms, dinoflagellates, desmids, and green algae. It has also been found to expand its diet from plant materials to include insects and fish [38]. The FAs composition reflects the composition of the diet, because “you are what you eat” [24]. Thus, expansion of diet and diversity of microalgae species contribute to* O. niloticus* having more types of *ω*3 PUFAs which are beneficial for the health of consumers as well as fish. The other three species,* L. niloticus*,* R. argentea*, and* T. zillii,* have specialized feeding as they increase in size.* L. niloticus* is a predator that feeds on fish (including its own species), crustaceans, and insects [39].* R. argentea* is zooplanktivorous feeding on zooplankton, surface insects, chironomids, and the prawns (*Caridina nilotica*) [40].* T. zillii* adults feed on phytoplankton, detritus, and macrophytes [41]. Due to their specialized feeding, they limit the diversity of food and therefore PUFAs compared to the omnivorous* O. niloticus*.

In addition, more types of *ω*3 PUFAs in* O. niloticus* could be as a result of desaturation and elongation of FAs. The ability to elongate and desaturate FAs is not the same in all species of fish.* O. niloticus* have the ability to bioconvert stearic acid, oleic acid, and other FAs, which belong to group C:18 FAs, to highly unsaturated FAs [14]. For example, arachidonic FA (20:4n-6) is a product of an elongation and desaturation of metabolic precursor of linoleic acid (18:2n-6), whereas EPA and DHA their metabolic precursor are alpha linolenic acid. Stearic acid, oleic acid, and other C:18 groups were found to be dominant FAs, contributing to higher composition of FAs in* O. niloticus*. By virtue of this capability, Muinde et al. [18] classified* O. niloticus* as an excellent source of *ω*3 PUFAs and being ideal for production of *ω*3 supplements.

The present study indicated that* R. argentea* have relatively higher levels of both EPA and DPA. This finding is contrary to Mwanja et al. [15] who found low levels of EPA in* R. argentea*. In this study the higher levels of EPA and DPA in* R. argentea* could be attributed to its feeding behavior.* R. argentea* utilizes zooplankton as its main food item [42]. EPA and DHA are very abundant in microalgae such as diatoms and dinoflagellates [43]. After consumption the acids move through the food chain unchanged via zooplankton to fish [44]. That is why copepods and cladoceran are also rich in EPA [45]. Adult* R. argentea* explore the bottom zone during daytime which is the habitat for zooplankton and macrobenthic invertebrates [46]. Furthermore, the relatively higher levels of EPA and DPA in* R. argentea* may also be attributed to the swimming mode and pattern.* R. argentea* is a slow swimmer commonly exhibiting vertical movements for avoidance of predators in search for food [47]. On the contrary,* O. niloticus, T. zillii*, and* L. niloticus* are fast swimmers utilizing more energy for movements against current. For this reason it is more likely for* R. argentea* to conserve more EPA and DPA, which account for higher levels of these FAs than those in the other species. Thus fish consumers should eat more* R. argentea* due to its high nutritional value and low selling price.

## 5. Conclusion

The current study identified 36 FAs in four commercial species of fish from Mwanza Gulf of Lake Victoria.* O*.* niloticus* was found to contain more types of FAs and *ω*3 PUFAs than* L. niloticus*,* T. zillii*, and* R. argentea*. Moreover,* R. argentea* has significantly and relatively higher levels of EPA and DPA than the other three commercial fish species from Mwanza Gulf of Lake Victoria. Thus fish consumers should eat more* O*.* niloticus* to get a variety of FAs types and* R. argentea* to obtain high levels of EPA and DPA.

## Figures and Tables

**Table 1:** Fatty acid composition from the four commercial fish species of Mwanza Gulf in Lake Victoria.

Fatty acid	*L. niloticus *	*O. niloticus *	*R. argentea *	*T. zillii *	Level of saturation
Myristic acid	∗	∗	∗	∗	Saturated
Pentadecanoic acid	∗	∗	∗	∗	Saturated
Palmitic acid	∗	∗	∗	∗	Saturated
Tricosanoic acid	∗	—	∗	—	Saturated
Stearic acid	∗	∗	∗	∗	Saturated
Heptadecanoic acid	∗	∗	∗	∗	Saturated
Lignoceric acid	∗	∗	∗	∗	Saturated
Nonadecanoic acid	∗	∗	∗	∗	Saturated
Heneicosanoate	∗	∗	—	∗	Saturated
Tetradecenoic acid	—	∗	—	—	MUFA
9-Octadecenoic acid	—	∗	—	—	MUFA
11-Octadecenoic acid	∗	∗	—	∗	MUFA
Tetradecanoate	—	∗	—	—	MUFA
Heptadecenoic acid	∗	∗	∗	∗	MUFA
Hexadecenoic acid	∗	∗	∗	∗	MUFA
11-Eicosenoic acid	—	∗	—	∗	MUFA
Oleic acid	∗	∗	∗	∗	MUFA
11,13-Eicosadienoic acid	—	∗	—	—	PUFA
11,14-Eicosadienoic acid	—	∗	∗	—	PUFA
Linoleic acid	∗	∗	∗	∗	PUFA
Arachidonic acid	∗	∗	∗	∗	PUFA
Eicosatrienoic acid	∗	∗	—	∗	PUFA
Docosatetraenoic acid	∗	∗	—	∗	PUFA
4,7,10,13,16-Docosapentaenoate	∗	∗	∗	∗	PUFA
Docosatrienoic acid	∗	∗	∗	∗	PUFA
Docosahexaenoic acid	∗	∗	∗	∗	PUFA
Alpha linolenic acid	—	∗	—	—	PUFA
Heneicosapentaenoic acid	∗	∗	—	∗	PUFA
Eicosatrienoic acid	∗	∗	—	∗	PUFA
Eicosapentaenoic acid	∗	∗	∗	∗	PUFA
Docosapentaenoic acid	∗	∗	—	∗	PUFA
Eicosatetraenoic acid	∗	∗	∗	∗	PUFA
8,11-Octadecadienoic acid	∗	∗	∗	∗	Unsaturated
10,13-Octadecadienoic acid	∗	—	∗	—	Unsaturated
Eicosadienoic acid	—	∗	—	—	Unsaturated
7,10-Hexadecadienoic acid	—	∗	—	—	Unsaturated

**Table 2:** Types of *ω*3 PUFAs found in each individual species.

Type of FA	*L. niloticus*	*O. niloticus *	*R. argentea *	*T. zillii *
Docosatrienoic acid	∗	∗	∗	∗
Docosahexaenoic acid	∗	∗	∗	∗
Alpha linolenic acid	—	∗	—	—
Heneicosapentaenoic acid	∗	∗	—	∗
Eicosatrienoic acid	∗	∗	—	∗
Eicosadienoic acid	—	∗	—	—
Eicosapentaenoic acid	∗	∗	∗	∗
Docosapentaenoic acid	∗	∗	—	∗
Eicosatetraenoic acid	∗	∗	∗	∗
